# Respiratory mechanic instability in evaluating the effectiveness of adenotonsillectomy for pediatric obstructive sleep apnea

**DOI:** 10.1016/j.jped.2025.101479

**Published:** 2025-12-03

**Authors:** Ji Ho Choi, Yeji Lee, Sungkyoung Shin, Tae Kyoung Ha, Se A. Lee

**Affiliations:** a Soonchunhyang University, College of Medicine, Bucheon Hospital, Department of Otorhinolaryngology-Head and Neck Surgery, Bucheon, South Korea; b Sungshin Women’s University, Department of Psychology, Seoul, South Korea; c Honeynaps Research and Development Center, Honeynaps Co. Ltd, Seoul, South Korea

**Keywords:** Obstructive sleep apnea, Children, Adenotonsillectomy, Respiratory mechanic instability, Polysomnography

## Abstract

**Objectives:**

Respiratory mechanic instability (RMI) is characterized by irregular chest and abdominal movements due to upper airway collapse or obstruction, frequently observed during obstructive apnea or hypopnea events. This study aims to explore the potential of RMI as a determinant of adenotonsillectomy's success in treating pediatric obstructive sleep apnea (OSA).

**Methods:**

Medical records of pediatric patients diagnosed with OSA via standard polysomnography who underwent adenotonsillectomy with subsequent follow-up evaluations were retrospectively analyzed. RMI parameters were automatically discerned during polysomnography, and comparisons of sleep scoring data, respiratory events, and RMI metrics (events, index, duration, and % of stage duration) were made pre- and post-surgery.

**Results:**

A total of 42 consecutive pediatric patients with OSA (26 boys, 16 girls) were included in the study. Post-adenotonsillectomy, marked improvements were noted in RMI parameters (events [95.6 ± 43.7 vs 58.9 ± 34.7; *p* < 0.001], index [14.5 ± 7.0 vs 8.5 ± 4.5; *p* < 0.001], duration [81.2 ± 56.3 vs 42.1 ± 52.0; *p* = 0.003], and % of stage duration [20.5 ± 13.9 vs 7.6 ± 6.8; *p* < 0.001]), along with significant improvements in polysomnographic parameters such as arousal index, AHI, RDI, ODI3, mean oxygen saturation, and snoring duration. Significant correlations were observed between respiratory events (AHI, RDI, ODI3) and RMI metrics such as event counts and index, both before and after the procedure.

**Conclusion:**

This study highlights the effectiveness of adenotonsillectomy in improving sleep quality and respiratory stability in pediatric patients with OSA, underscoring the utility of RMI as a promising additional marker in conjunction with conventional polysomnographic measures.

## Introduction

Obstructive sleep apnea (OSA) is a common disorder affecting pediatric populations, characterized by repeated episodes of partial or complete upper airway obstruction during sleep.^1^ It is estimated that OSA impacts approximately 1–5 % of pediatric individuals, with its peak incidence occurring between the ages of 2 and 8 years — a period that commonly coincides with adenotonsillar tissues reaching their maximum size relative to the dimensions of the airway.^2^ These recurrent episodes of airway collapse result in intermittent hypoxia, hypercapnia, and disrupted sleep patterns, which are associated with substantial clinical signs, physiological complications, and developmental impacts.^3^^,^^4^ The manifestations of pediatric OSA go beyond nighttime symptoms such as snoring, gasping, and apneic pauses, to include a variety of daytime impairments like behavioral issues, cognitive deficits, and cardiovascular problems.^5^, ^6^, ^7^, ^8^ If left untreated, OSA in children can lead to long-term consequences, including developmental delays and an elevated risk of hypertension and metabolic syndrome later in life.^7^, ^8^, ^9^ Considering these extensive effects, it is crucial to manage pediatric OSA promptly and effectively.^10^ Adenotonsillectomy, the surgical excision of the adenoids and tonsils, has long been recognized as the standard treatment for pediatric OSA, primarily because adenotonsillar hypertrophy is a major anatomical factor in the development of this condition in children.^11^ Numerous studies have confirmed the effectiveness of adenotonsillectomy in alleviating the severity of OSA and enhancing the quality of life.^12^^,^^13^ Despite its common application, the outcomes of adenotonsillectomy vary, and a significant number of patients still exhibit residual symptoms or respiratory issues after surgery.^14^^,^^15^

Respiratory mechanic instability (RMI) refers to the paradoxical or abnormal movement of the chest and abdomen stemming from upper airway occlusion or narrowing, as observed in conditions such as apnea or hypopnea.^16^, ^17^, ^18^ RMI was identified by analyzing inductance plethysmography data to quantify variations in the phase relationship between thoracic and abdominal respiratory motions. The parameters of RMI include four key metrics: RMI events, RMI index, RMI duration, and % of stage duration. Previous studies have demonstrated a significant correlation between RMI parameters and respiratory parameters like the apnea-hypopnea index (AHI) and oxygen desaturation index ≥ 3 % (ODI3), noted in both adults and children.^16^^,^^18^ Additionally, the area under the receiver operating characteristic curve (AUROC) was calculated to evaluate the diagnostic performance of RMI parameters, demonstrating their capability to distinguish between OSA patients and controls, and to differentiate among various OSA subgroups.^16^^,^^18^ Recent studies indicate that RMI parameters show significant improvement following successful therapy in adult patients with OSA, highlighting its potential as a valuable tool for assessing the efficacy of treatments such as positive airway pressure (PAP).^17^ Nevertheless, the effectiveness of RMI in evaluating post-treatment outcomes in pediatric patients with OSA remains undetermined. Therefore, this study aims to establish whether RMI can serve as a viable measure for evaluating the effectiveness of adenotonsillectomy in treating pediatric OSA.

## Methods

### Subjects

This study was approved by the Institutional Review Board at Soonchunhyang University Bucheon Hospital following a detailed review process (IRB No 2025–02–007). The medical records of pediatric patients diagnosed with OSA were retrospectively gathered for the analysis, spanning from January 2017 to December 2024. The inclusion criteria specified pediatric patients aged 18 years or younger who satisfied the following conditions: (1) a diagnosis of OSA established through attended, in-laboratory, overnight standard polysomnography (level-I), recognized as the definitive diagnostic method for OSA; (2) their consent to undergo an adenotonsillectomy as the chosen intervention for their disorder; and (3) they completed a follow-up assessment with standard polysomnography (level-I) post-surgery.^19^ The exclusion criteria for this study included patients who met any of the following conditions: (1) those diagnosed with central sleep apnea syndrome, neuromuscular disorders, or other co-occurring sleep disorders, such as periodic limb movement disorder, potentially impacting study outcomes; and (2) those whose medical records were missing, incomplete, or unavailable for thorough and precise analysis.

### Polysomnography

All participants in the study underwent comprehensive, full-night standard polysomnography (level-I) both before and after their surgical interventions. A follow-up polysomnography (PSG) was conducted approximately 3 months postoperatively. The polysomnography tests employed a detailed setup that monitored multiple parameters: electroencephalography (EEG) to record brain activity, electrooculography (EOG) channels to track eye movements, and electrocardiography (ECG) to monitor heart function. Additionally, electromyography (EMG) measured muscle activity, including that of the chin and bilateral anterior tibialis muscles. Airflow through the nose and mouth was assessed, and respiratory effort was detected using sensors placed on the chest and abdomen, while blood oxygen saturation was continuously monitored via oximetry. A trained sleep technician meticulously observed and documented the patients’ behaviors throughout the sleep study using an infrared camera installed in the monitoring room. Data from the polysomnography were collected using the digitalized system (Embla N7000/Embletta MPR, Medcare-Embla, Reykjavik, Iceland) to ensure high-quality recordings. All data were meticulously scored manually by the sleep technician according to the American Academy of Sleep Medicine (AASM) scoring manual.^20^ Following this initial scoring, the data were meticulously reviewed by certified physicians to confirm accuracy and consistency. The AHI is defined as the total number of all respiratory events (obstructive, central, and mixed apneas and hypopneas) per hour of Total Sleep Time (TST). In contrast, the obstructive AHI (OAHI) is defined as the count of only the obstructive and mixed apneas and obstructive hypopneas per hour of TST.

### Respiratory mechanic instability

The RMI was calculated by analyzing changes or variations in the phase relationship between thoracoabdominal respiratory effort signals. This assessment involved evaluating the alignment or misalignment (thoraco-abdominal asynchrony [TAA]) of these signals during the respiratory cycle. A baseline reference of a 10-degree phase difference was established for these measurements, providing a standard against which deviations were compared. Thoracic and abdominal respiratory efforts were monitored continuously using XactTrace™ effort belts and inductance plethysmography technology. Crucially, the RMI measurement utilized only the signals obtained from the standard chest and abdominal respiratory effort belts used in routine polysomnography, requiring no additional sensors or specialized equipment. Artifacts were carefully identified and removed from the recorded data. Additionally, periods representative of stage W, denoting wakefulness, were excluded from the analysis. RMI events were characterized by significant phase variations (≥ 10 degrees) between the thorax and abdominal respiratory effort signals. The RMI index was calculated by determining the frequency of these events per hour of total sleep time (TST). RMI duration was expressed in minutes and referred to the period of marked respiratory instability that exceeded the established baseline. Furthermore, the percentage of RMI in stage duration represented the proportion of time spent in RMI during each specific sleep stage. In this study, RMI values were derived by calculating the mean across all analyzed sleep stages. For a detailed description and visual explanation of the RMI analysis method, including the assessment of thoracoabdominal synchrony and paradoxical movement, please refer to our prior publication.^16^

### Surgery

The adenotonsillectomy procedure was performed by an experienced surgeon (JHC) under general anesthesia as a standard clinical intervention for patients diagnosed with OSA.^21^ The adenoidectomy was carried out using a microdebrider and guided by endovisual systems, which enabled precise and clear visualization.^22^ Bipolar forceps were used during the tonsillectomy to meticulously remove the tonsils. Transient packing gauze was applied to the adenoidectomy site to control bleeding and ensure effective hemostasis, remaining in place for 5 to 10 min. The hemostasis at the tonsillectomy site was successfully managed with bipolar forceps, resulting in minimal blood loss throughout the procedure.

### Statistical analysis

Results were presented as the mean ± standard deviation (SD) for continuous variables, offering a concise summary of the data distribution. A two-tailed paired *t*-test was employed to compare preoperative and postoperative polysomnography and RMI parameters. Pearson correlation analysis determined the strength and direction of associations between two continuous variables. The statistical analyses were performed using R software (version 3.4.3; The R Foundation for Statistical Computing, Vienna, Austria), with a p-value of <0.05 deemed statistically significant.

## Results

This study consecutively enrolled forty-two children with OSA (26 boys, 16 girls) who underwent standard, full-night polysomnography both before and after adenotonsillectomy. The children's average age ± SD was 7.3 ± 3.2 years, and the average BMI ± SD was 19.6 ± 4.9. Based on BMI percentile classifications, underweight (< 5th percentile) was observed in 1 participant (2.38 %), healthy weight (5th ≤ BMI < 85th percentile) in 21 participants (50.0 %), overweight (85th ≤ BMI < 95th percentile) in 8 participants (19.05 %), and obesity (≥ 95th percentile) in 12 participants (28.57 %).

The comparison of sleep scoring data in children with OSA before and after adenotonsillectomy is presented in Table 1. No significant changes were observed in total recording time (TRT: 467.6 ± 53.2 min vs. 461.3 ± 41.5 min, *p* = 0.451) or total sleep time (TST: 401.4 ± 54.9 min vs. 410.2 ± 48.9 min, *p* = 0.314). Additionally, there were no significant differences in sleep latency (17.0 ± 13.2 min vs. 14.4 ± 15.1 min, *p* = 0.360) and wake after sleep onset (WASO: 48.1 ± 30.1 min vs. 36.7 ± 23.9 min, *p* = 0.178). Sleep efficiency showed a slight, nonsignificant increase from 86.2 % ± 9.6 % to 88.9 % ± 7.0 % (*p* = 0.151). Significant improvements were noted in the arousal index, which decreased from 18.0 ± 10.7 to 10.1 ± 5.5 events per hour (*p* < 0.001). Stage N1 sleep decreased significantly from 9.2 % ± 5.5 % to 6.3 % ± 4.4 % (*p* < 0.001). Conversely, Stage N2 sleep showed no significant change (38.1 % ± 10.1 % to 40.4 % ± 8.4 %, *p* = 0.684), and Stage N3 sleep exhibited a slight, non-significant increase from 31.9 % ± 12.0 % to 32.7 % ± 10.8 % (*p* = 0.409). In contrast, the proportion of REM sleep significantly increased from 15.2 % ± 5.3 % to 17.6 % ± 4.3 % (*p* = 0.03).

**Table 1:** Comparison of sleep scoring data pre- and post-adenotonsillectomy in children diagnosed with obstructive sleep apnea.

Sleep scoring data	Before	After	p-value
Total recording time (TRT), min	467.6 ± 53.2	461.3 ± 41.5	0.451
Total sleep time (TST), min	401.4 ± 54.9	410.2 ± 49.9	0.314
Sleep onset, min	17.0 ± 13.2	14.4 ± 15.1	0.360
Wake after sleep onset(WASO), min	49.1 ± 50.1	36.7 ± 29.9	0.173
Sleep efficiency, %	86.2 ± 9.6	88.9 ± 7.0	0.151
Arousal index, events/hour of TST	18.0 ± 10.7	10.1 ± 5.5	<0.001
Stage N1, % of TST	9.8 ± 5.5	6.3 ± 4.1	<0.001
Stage N2, % of TST	39.1 ± 10.1	40.4 ± 8.4	0.654
Stage N3, % of TST	31.9 ± 12.0	32.72 ± 10.3	0.409
Stage R, % of TST	15.2 ± 5.3	17.6 ± 4.3	0.03

Data are presented as mean ± standard deviation. N1, non-rapid eye movement (REM) sleep stage 1; N2, non-REM sleep stage 2; N3, non-REM sleep stage 3; R, REM sleep stage.

Table 2 illustrates the comparison of respiratory events in children with OSA before and after adenotonsillectomy. There was a significant decrease in the average AHI, from 18.1 ± 30.3 to 2.2 ± 2.0 events/hour post-surgery (*p* < 0.001). Central apnea (CA) exhibited a non-significant reduction from 1.1 ± 1.0 to 0.8 ± 1.1 events/hour (*p* = 0.166). The Respiratory Disturbance Index (RDI) significantly dropped from 21.0 ± 29.5 to 3.1 ± 3.5 events/hour (*p* < 0.001), and the ODI3 significantly decreased from 17.2 ± 30.7 to 2.6 ± 2.8 events/hour (*p* = 0.003). Mean oxygen saturation improved slightly but significantly from 96.5 % ± 1.6 % to 97.2 % ± 0.8 % (*p* = 0.008). The lowest oxygen saturation increased from 84.4 % ± 15.6 % to 88.5 % ± 14.3 %, although this change was not statistically significant (*p* = 0.223). Additionally, snoring time was significantly reduced from 19.1 % ± 19.6 % to 5.9 % ± 16.1 % (*p* = 0.002).

**Table 2:** Comparison of respiratory events pre- and post-adenotonsillectomy in children with obstructive sleep apnea.

Respiratory events	Before	After	p-value
Apnea-hypopnea index (AHI), events/hour of TST	18.1 ± 30.3	2.2 ± 2.0	0.001
Central apnea (CA), events/hour of TST	1.1 ± 1.0	0.8 ± 1.1	0.166
Respiratory disturbance index (RDI), events/hour of TST	21.0 ± 29.5	3.1 ± 3.5	<0.001
Oxygen desaturation index ≥ 3 % (ODI3), events/hour of TST	17.2 ± 30.7	2.6 ± 2.8	0.003
Mean oxygen saturation, %	96.5 ± 1.6	97.2 ± 0.8	0.008
Lowest oxygen saturation, %	84.4 ± 15.6	88.5 ± 14.3	0.223
Snoring, % of TST	19.1 ± 19.6	5.9 ± 16.1	0.002

Data are presented as mean ± standard deviation. TST, total sleep time.

Table 3 displays the comparison of RMI parameters in children with OSA before and after adenotonsillectomy. The number of RMI events significantly declined from 95.6 ± 43.7 to 58.9 ± 34.7 after surgery (*p* < 0.001). Surgical intervention led to a substantial reduction in the RMI index, with values decreasing significantly from 14.5 ± 7.0 to 8.5 ± 4.5 (*p* < 0.001). Postoperatively, the average duration of RMI episodes significantly decreased to 42.1 ± 52.0 min from a pre-operative average of 81.2 ± 56.3 min (*p* = 0.003). The percentage of sleep time spent in RMI stages significantly decreased from 20.5 % ± 13.9 % before surgery to 7.6 % ± 6.8 % after surgery (*p* < 0.001).

**Table 3:** Comparison pre- and post-adenotonsillectomy of respiratory mechanical instability (RMI) parameters in children with obstructive sleep apnea.

Respiratory mechanics instability (RMI) parameters	Before	After	p-value
Events	95.6 ± 43.7	58.9 ± 34.7	<0.001
Index, events/hour of TST	14.5 ± 7.0	8.5 ± 4.5	<0.001
Duration, min	81.2 ± 56.3	42.1 ± 52.0	0.003
Stage duration, %	20.5 ± 13.9	7.6 ± 6.8	<0.001

Data are presented as mean ± standard deviation.

**Fig. 1:**
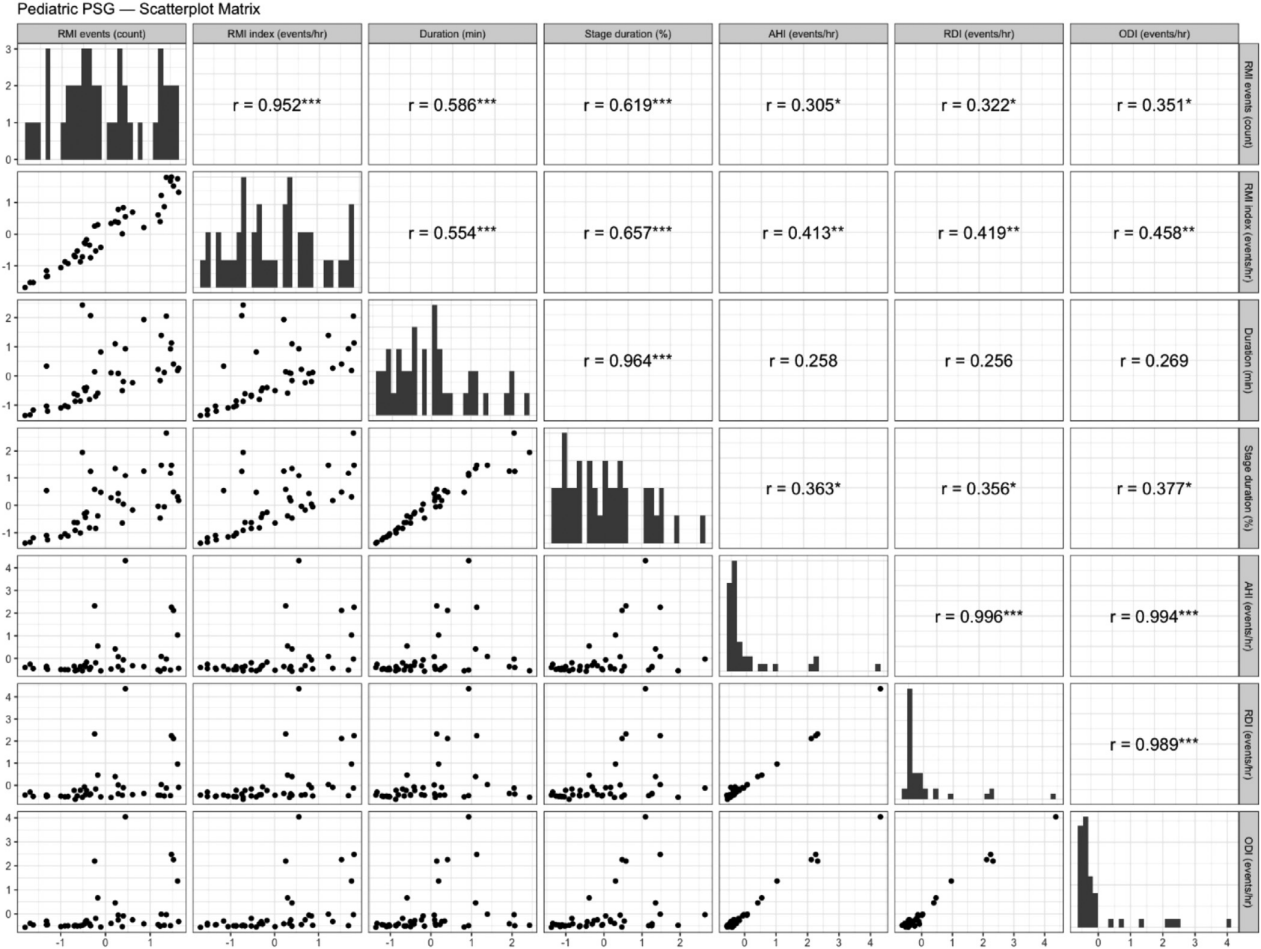
Scatterplot matrix illustrating the correlations between respiratory mechanical instability parameters and respiratory events in pediatric patients with obstructive sleep apnea prior to adenotonsillectomy. All variables were standardized (z-scored: mean = 0, standard deviation = 1) to eliminate differences in measurement scales, with both the x and y axes represented in standardized units. The diagonal panels show the histogram of each standardized variable's distribution. The lower panels display pairwise scatterplots illustrating the correlation between each pair of variables. The upper panels indicate the Pearson’s correlation coefficients (r) along with their significance levels (*p < 0.05, **p < 0.01, ***p < 0.001).

**Fig. 2:**
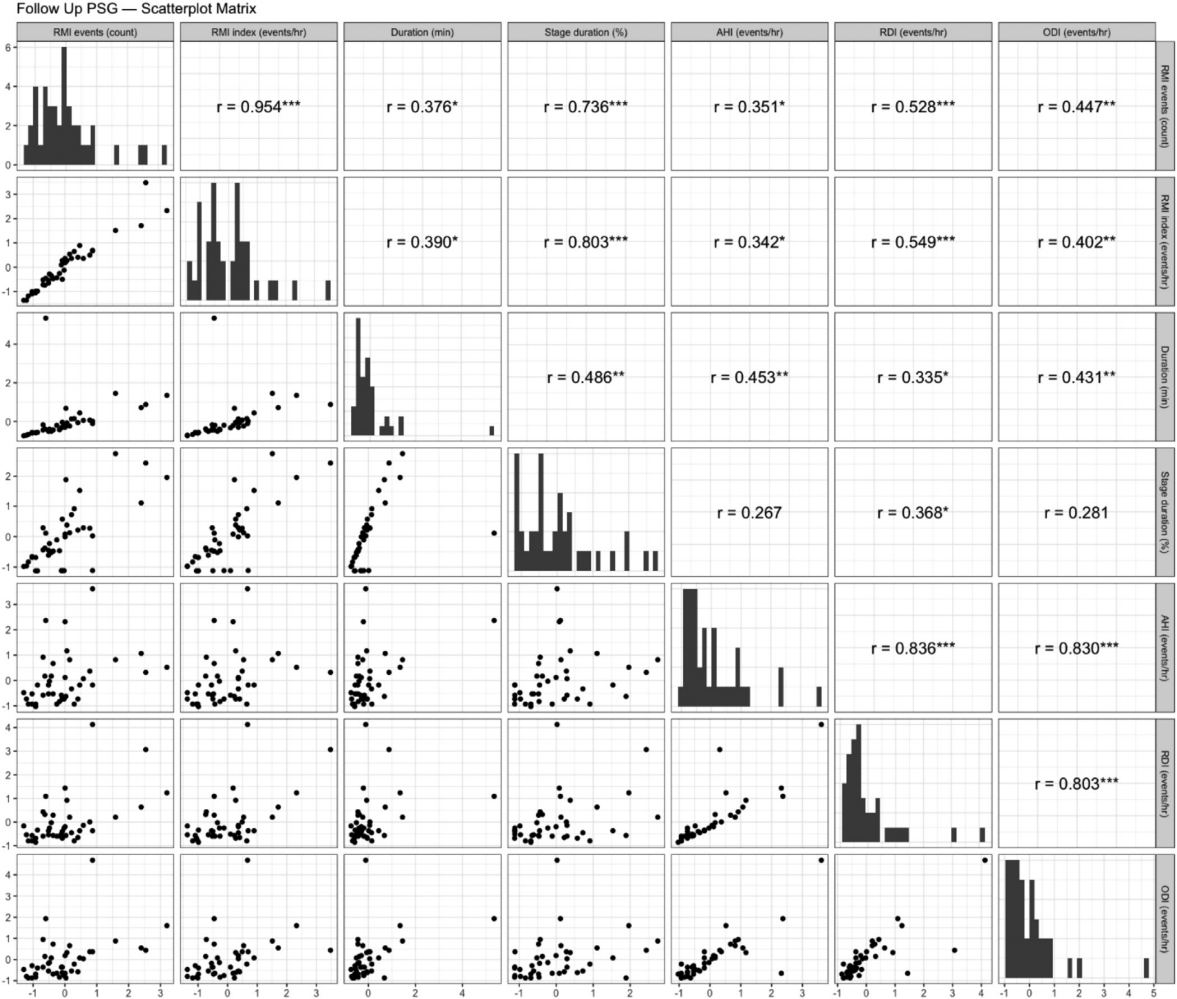
Scatterplot matrix illustrating the correlations between respiratory mechanical instability parameters and respiratory events in pediatric patients with obstructive sleep apnea following adenotonsillectomy. All variables were standardized (z-scored: mean = 0, standard deviation = 1) to eliminate differences in measurement scales, with both the x and y axes represented in standardized units. The diagonal panels show the histogram of each standardized variable's distribution. The lower panels display pairwise scatterplots illustrating the correlation between each pair of variables. The upper panels indicate the Pearson’s correlation coefficients (r) along with their significance levels (*p < 0.05, **p < 0.01, ***p < 0.001).

Correlations between RMI parameters and respiratory events in pediatric patients with OSA pre- and post-adenotonsillectomy are depicted in Fig. 1, Fig. 2. Before adenotonsillectomy, significant moderate correlations were observed between RMI events and AHI (*r* = 0.305, *p* < 0.05), RDI (*r* = 0.322, *p* < 0.05), and ODI (*r* = 0.351, *p* < 0.05). These significant associations persisted postoperatively with AHI (*r* = 0.351, *p* < 0.05), RDI (*r* = 0.528, *p* < 0.001), and ODI (*r* = 0.447, *p* < 0.01). The RMI index also demonstrated significant moderate correlations with AHI (*r* = 0.413, *p* < 0.01), RDI (*r* = 0.419, *p* < 0.05), and ODI (*r* = 0.458, *p* < 0.05) prior to surgery, with these significant associations continuing post-surgery for AHI (*r* = 0.342, *p* < 0.05), RDI (*r* = 0.549, *p* < 0.001), and ODI (*r* = 0.402, *p* < 0.05). Post-surgery, the duration of RMI events correlated significantly and moderately with AHI (*r* = 0.453, *p* < 0.01), RDI (*r* = 0.335, *p* < 0.05), and ODI (*r* = 0.431, *p* < 0.01), a relationship not observed before surgery. The percentage of sleep time in RMI stages showed significant moderate pre-surgery correlations with AHI (*r* = 0.363, *p* < 0.05), RDI (*r* = 0.356, *p* < 0.05), and ODI (*r* = 0.377, *p* < 0.05), maintaining statistical significance post-surgery for RDI (*r* = 0.368, *p* < 0.05).

## Discussion

This study underscores the effectiveness of RMI as a valuable tool for assessing the outcomes of adenotonsillectomy in pediatric OSA patients. The key findings are summarized as follows: Adenotonsillectomy resulted in significant improvements in the arousal index, stage N1, and stage R; however, changes in TST, sleep efficiency, and WASO were not statistically significant. Respiratory metrics, including AHI, RDI, ODI3, mean oxygen saturation, and snoring duration, demonstrated substantial postoperative enhancement, but central apnea and lowest oxygen saturation showed no change. RMI parameters such as event frequency, index, duration, and stage duration significantly decreased after the procedure, indicating enhanced respiratory stability in pediatric OSA. Moreover, significant moderate correlations were observed between respiratory events and RMI parameters pre- and post-adenoidectomy in most instances. Based on obstructive respiratory events, 30 of 42 children with OSA (71.4 %) normalized post-adenotonsillectomy (AHI < 1). Mild sleep apnea (1 ≤ AHI < 5) persisted in 10 patients (23.8 %), and moderate OSA (5 ≤ AHI < 10) in 2 patients (4.8 %). Notably, 37 out of 42 (88.1 %) patients achieved an AHI of <2 post-surgery.

The significant reduction in arousal index post-surgery (*p* < 0.001) illustrates a decrease in sleep fragmentation, a critical enhancement for overall sleep quality. These changes are linked to a reduction in arousals due to respiratory events. A notable shift in sleep stage proportions, with a significant decrease in N1 sleep (*p* < 0.001) and an increase in REM sleep (*p* = 0.03), points to a normalization of sleep architecture towards a more restorative pattern. However, no significant changes were observed in stages N2 and N3. These intriguing results may suggest a more stable sleep architecture in children compared to adults, particularly regarding respiratory events, or reflect the partial impact of the surgical intervention for pediatric OSA.^5^^,^^23^

The substantial improvements observed in traditional respiratory metrics, such as the AHI and the RDI (*p* < 0.001 for both), robustly confirm the positive impact of adenotonsillectomy in mitigating obstructive respiratory disturbances. Additionally, the meaningful decrease in the ODI3 and the rise in mean oxygen saturation (*p* = 0.003 and *p* = 0.008, respectively) further underscore the enhancements in oxygenation and respiratory function during sleep. However, the increase in the lowest oxygen saturation ( %) from 84.4 ± 15.6 to 88.5 ± 14.3 was not statistically significant, likely stemming from the substantial variability (high standard deviation) in the data. Moreover, the significant reduction in snoring time (*p* = 0.002) contributes to a more restful sleep environment. However, central apnea did not show significant changes post-surgery in this study, an expected finding given the characteristics of RMI in the presence of obstructive respiratory disturbances.^16^, ^17^, ^18^

The most compelling evidence of adenotonsillectomy's efficacy, and a pivotal contribution of this study, comes from marked improvements in RMI parameters. The significant reduction in the number of RMI events (*p* < 0.001) and the notable decrease in the RMI index (*p* < 0.001) clearly demonstrate enhanced respiratory stability. These results are substantiated by the significant decrease in the duration of RMI episodes (*p* = 0.003) and the dramatic reduction in the percentage of sleep time spent in RMI states (*p* < 0.001). These findings pertaining to RMI may provide a deeper insight into the surgical benefits, spotlighting improvements in respiratory events and reduced tendency toward respiratory instability beyond what is measurable by traditional AHI and RDI metrics. Moreover, although not fully elucidated, the RMI parameter may offer a more sensitive indicator of overall respiratory health and stability during sleep by capturing instabilities that may not present as distinct apnea or hypopnea events. Further research in this area is essential.

The observed significant correlations between RMI parameters and conventional respiratory indices such as AHI, RDI, and ODI3, both pre- and post-adenotonsillectomy, suggest that RMI may serve as an additional useful measure for assessing respiratory events in pediatric OSA. Notably, among the RMI parameters, RMI events, and the index exhibited significant correlations with all three respiratory indices, AHI, RDI, and ODI3, both pre- and post-surgery. RDI demonstrated a relatively high correlation with RMI parameters, with the strongest correlation observed between the postoperative RMI index and RDI (*r* = 0.549, *p* < 0.001). This likely stems from RDI's inclusion of not only apneas and hypopneas but also respiratory effort-related arousals (RERAs), which may more accurately reflect the subtle respiratory instabilities captured by RMI parameters. However, as no studies to date have investigated the association between RMI parameters and RERAs, future research should address these questions.

Several studies have examined the association between RMI parameters and OSA.^16^, ^17^, ^18^ Choi et al. assessed RMI as a diagnostic tool and its correlation with other polysomnographic parameters in 189 adult patients with OSA.16 The findings revealed that RMI parameters significantly correlated with AHI, arousal index, and oxygen desaturation index, demonstrating strong diagnostic performance in predicting OSA and its subgroups. Furthermore, the authors explored the use of RMI parameters in evaluating the efficacy of positive airway pressure (PAP) therapy in OSA treatment.17 The results indicated that all RMI parameters significantly improved following the alleviation of obstructive respiratory disturbance parameters (AHI, ODI3, and lowest oxygen saturation), as evidenced by the comparison of diagnostic polysomnography and PAP titration data. In 2022, Ryu et al. investigated the relationships between RMI parameters and polysomnographic indices and assessed the utility of RMI in diagnosing OSA in children.18 Among 263 children tested, 183 (70.4 %) were diagnosed with OSA, and RMI parameters were elevated and showed correlation with respiratory events, especially in moderate or severe OSA without central apnea, yielding AUROCs above 0.65, the highest being for the percentage of RMI in stage duration.

Both the Liu et al. study and the present study share a common conceptual framework, focusing on the assessment of TAA as an indicator of inspiratory effort or respiratory instability in pediatric obstructive sleep apnea.^24^ However, Liu et al. quantified TAA by analyzing instantaneous phase differences between ribcage and abdominal excursions during artifact-free, non-event epochs of polysomnography, averaging these values across sleep stages. In contrast, this study employed automated signal analysis using XactTrace™ inductance belts to calculate respiratory phase variability beyond a 10-degree threshold, defining multiple quantitative indices (event count, index, duration, and stage-specific percentage) to characterize respiratory mechanical instability in greater detail. Therefore, RMI provides unique information distinct from conventional chest and abdominal effort signals; it is not merely a repackaging of existing data but a metric that captures unquantified physiological phenomena, thus adding novel diagnostic value.

The limitations of this study include its retrospective design, a relatively small sample size that was not determined by formal calculations, and the challenge of generalizing the findings. Although RMI is calculated automatically from standard polysomnography effort belts (XactTraceTM) using the Embla systems, its practical utility is limited by its current dependency on vendor-specific equipment and software algorithms. Future studies are expected to address these limitations.

## Conclusion

This study demonstrates the effectiveness of adenotonsillectomy in improving sleep quality and respiratory stability in children with OSA, utilizing not only traditional polysomnographic parameters but also RMI metrics. Including RMI assessment results in a more comprehensive evaluation of surgical outcomes in pediatric OSA. This highlights the utility of RMI as a sensitive and promising additional marker of improved respiratory mechanics and stability post-adenotonsillectomy. Future research should delve into the clinical implications of RMI parameters and their potential in guiding personalized treatment strategies for pediatric OSA.

## Availability of data and material

The datasets used and/or analyzed during the current study may be provided by the corresponding author, upon appropriate request.

## Funding source

This work was supported by the Technology Development Program (RS-2023–00321754) funded by the Ministry of SMEs and Startups (MSS, Korea). This study was supported by the Soonchunhyang University Research Fund. However, these funding sources had no involvement in the study design, collection, analysis, and interpretation of data, writing of the report, and the decision.

## CRediT authorship contribution statement

**Ji Ho Choi:** Conceptualization, Data curation, Formal analysis, Funding acquisition, Investigation, Supervision, Writing – original draft, Writing – review & editing. **Yeji Lee:** Investigation. **Sungkyoung Shin:** Formal analysis, Investigation. **Tae Kyoung Ha:** Funding acquisition, Investigation. **Se A. Lee:** Supervision, Writing – review & editing.

## Conflicts of interest

The authors declare no conflicts of interest.

## References

[1] Guilleminault C., Abad V.C. (2004). Obstructive sleep apnea. Curr Treat Options Neurol.

[2] Au C.T., Li A.M. (2009). Obstructive sleep breathing disorders. Pediatr Clin North Am.

[3] Halbower A.C., Marcus C.L. (2003). Sleep disorders in children. Curr Opin Pulm Med.

[4] Park D.Y., Choi J.H., Kang S.Y., Han J., Park H.Y., Hwang J.S. (2018). Correlations between pediatric obstructive sleep apnea and longitudinal growth. Int J Pediatr Otorhinolaryngol.

[5] Choi J.H., Kim E.J., Choi J., Kwon S.Y., Kim T.H., Lee S.H. (2010). Obstructive sleep apnea syndrome: a child is not just a small adult. Ann Otol Rhinol Laryngol.

[6] Owens J.A. (2009). Neurocognitive and behavioral impact of sleep disordered breathing in children. Pediatr Pulmonol.

[7] Capdevila O.S., Kheirandish-Gozal L., Dayyat E., Gozal D. (2008). Pediatric obstructive sleep apnea: complications, management, and long-term outcomes. Proc Am Thorac Soc.

[8] Arens R., Marcus C.L. (2004). Pathophysiology of upper airway obstruction: a developmental perspective. Sleep.

[9] Kwok K.L., Ng D.K., Chan C.H. (2008). Cardiovascular changes in children with snoring and obstructive sleep apnoea. Ann Acad Med Singap.

[10] Marcus C.L., Brooks L.J., Draper K.A., Gozal D., Halbower A.C., Jones J. (2012). Diagnosis and management of childhood obstructive sleep apnea syndrome. Pediatrics.

[11] Choi J.H., Oh J.I., Kim T.M., Yoon H.C., Park I.H., Kim T.H. (2015). Long-term subjective and objective outcomes of adenotonsillectomy in Korean children with obstructive sleep apnea syndrome. Clin Exp Otorhinolaryngol.

[12] Brietzke S.E., Gallagher D. (2006). The effectiveness of tonsillectomy and adenoidectomy in the treatment of pediatric obstructive sleep apnea/hypopnea syndrome: a meta-analysis. Otolaryngol Head Neck Surg.

[13] Todd C.A., Bareiss A.K., McCoul E.D., Rodriguez K.H. (2017). Adenotonsillectomy for obstructive sleep apnea and Quality of life: systematic review and meta-analysis. Otolaryngol Head Neck Surg.

[14] Bhattacharjee R., Kheirandish-Gozal L., Spruyt K., Mitchell R.B., Promchiarak J., Simakajornboon N. (2010). Adenotonsillectomy outcomes in treatment of obstructive sleep apnea in children: a multicenter retrospective study. Am J Respir Crit Care Med.

[15] Kang K.T., Hsu W.C. (2024). Efficacy of adenotonsillectomy on pediatric obstructive sleep apnea and related outcomes: a narrative review of current evidence. J Formos Med Assoc.

[16] Choi J.H., Lee B., Hwang S.H (2019). Association of Respiratory mechanic instability and Respiratory parameters among adults with obstructive sleep apnea. Otolaryngol Head Neck Surg.

[17] Choi J.H., Jung J.Y., Moon J.E., Hwang S.H. (2020). The usefulness of Respiratory mechanic instability in evaluating the effect of continuous positive airway pressure for obstructive sleep apnea. Ann Otol Rhinol Laryngol.

[18] Ryu G., Kim H.Y., Choi J.H. (2022). Associations of respiratory mechanic instability with respiratory parameters in pediatric patients with obstructive sleep apnea syndrome. Int J Pediatr Otorhinolaryngol.

[19] American Academy of Sleep Medicine (2014).

[20] Troester M.M., Quan S.F., Berry R.B., Plante D.T., Abreu A.R., Alzoubaidi M. (2023).

[21] Choi J.H., Kim E.J., Choi J., Kim T.H., Kwon S.Y., Lee S.H. (2009). The effect of adenotonsillectomy on changes of position during sleep in pediatric obstructive sleep apnea syndrome. Am J Rhinol Allergy.

[22] Kim J.W., Kim H.J., Lee W.H., Kim D.K., Kim S.W., Kim Y.H. (2015). Comparative study for efficacy and safety of adenoidectomy according to the surgical method: a prospective multicenter study. PLoS One.

[23] Alsubie H.S., BaHammam A.S. (2017). Obstructive sleep apnoea: children are not little adults. Paediatr Respir Rev.

[24] Liu X., Immanuel S., Pamula Y., Kennedy D., Martin J., Baumert M. (2017). Adenotonsillectomy for childhood obstructive sleep apnoea reduces thoraco-abdominal asynchrony but spontaneous apnoea-hypopnoea index normalisation does not. Eur Respir J.

